# Behavioral characterization of mice lacking *Trek* channels

**DOI:** 10.3389/fnbeh.2012.00060

**Published:** 2012-09-07

**Authors:** Kelsey Mirkovic, Jaime Palmersheim, Florian Lesage, Kevin Wickman

**Affiliations:** ^1^Department of Pharmacology, University of MinnesotaMinneapolis, MN, USA; ^2^Institut de Pharmacologie Moléculaire et Cellulaire Labex ICST, Université de Nice Sophia AntipolisValbonne, France

**Keywords:** knockout, potassium channel, behavior, morphine, anxiety, memory

## Abstract

Two-pore domain K^+^ (K_2P_) channels are thought to underlie background K^+^ conductance in many cell types. The Trek subfamily of K_2P_ channels consists of three members, *Trek1*/*Kcnk2*, *Trek2*/*Kcnk10*, and *Traak*/*Kcnk4*, all three of which are expressed in the rodent CNS. Constitutive ablation of the *Trek1* gene in mice correlates with enhanced sensitivity to ischemia and epilepsy, decreased sensitivity to the effects of inhaled anesthetics, increased sensitivity to thermal and mechanical pain, and resistance to depression. While the distribution of *Trek2* mRNA in the CNS is broad, little is known about the relevance of this *Trek* family member to neurobiology and behavior. Here, we probed the effect of constitutive *Trek2* ablation, as well as the simultaneous constitutive ablation of all three *Trek* family genes, in paradigms that assess motor activity, coordination, anxiety-related behavior, learning and memory, and drug-induced reward-related behavior. No differences were observed between *Trek2*^−/−^ and *Trek1/2/Traak*^−/−^ mice in coordination or total distance traveled in an open-field. A gender-dependent impact of *Trek* ablation on open-field anxiety-related behavior was observed, as female but not male *Trek2*^−/−^ and *Trek1/2/Traak*^−/−^ mice spent more time in, and made a greater number of entries into, the center of the open-field than wild-type counterparts. Further evaluation of anxiety-related behavior in the elevated plus maze and light/dark box, however, did not reveal a significant influence of genotype on performance for either gender. Furthermore, *Trek*^−/−^ mice behaved normally in tests of learning and memory, including contextual fear conditioning and novel object recognition, and with respect to opioid-induced motor stimulation and conditioned place preference (CPP). Collectively, these data argue that despite their broad distribution in the CNS, Trek channels exert a minimal influence on a wide-range of behaviors.

## Introduction

Two-pore domain K^+^ (K_2P_) channels are thought to underlie the background K^+^ conductance in many cell types (Enyedi and Czirjak, 2010). The K_2P_ channel family is diverse, with six sub-families containing members that differ primarily in terms of biophysical properties and regulation (Enyedi and Czirjak, 2010). The *Trek* subfamily of K_2P_ channels consists of three members—*Trek1*/*Kcnk2*, *Trek2*/*Kcnk10*, and the more distantly-related member, *Traak*/*Kcnk4*. Trek1 and Trek2 channel activity is regulated by a diverse set of stimuli including arachidonic acid (Patel et al., 1998; Lesage et al., 2000; Maingret et al., 2000), membrane stretch (Patel et al., 1998; Bang et al., 2000; Lesage et al., 2000), intracellular acidification (Maingret et al., 1999; Lesage et al., 2000; Kim et al., 2001), and heat (Maingret et al., 2000; Kang et al., 2005). Additionally, both Trek1 and Trek2 channels are inhibited by protein kinase A (PKA) and protein kinase C (PKC) phosphorylation, which couples these channels to G_s_, G_i/o_, and G_q_ G protein signaling cascades (Fink et al., 1996; Patel et al., 1998; Lesage et al., 2000; Maingret et al., 2000; Bockenhauer et al., 2001; Murbartian et al., 2005).

*In situ* hybridization revealed a broad distribution of *Trek1* in the rat CNS (Talley et al., 2001; Gu et al., 2002), and genetic ablation of *Trek1* correlates with multiple neurophysiological and neurobehavioral phenotypes. *Trek1*^−/−^ mice are more sensitive to ischemia and epilepsy, show lower sensitivity to the effects of inhaled anesthetics, and display an increased sensitivity to thermal and mechanical pain (Heurteaux et al., 2004; Alloui et al., 2006; Noel et al., 2009). *Trek1*^−/−^ mice also exhibit a depression-resistant phenotype, suggesting that Trek1-containing channels are a potential downstream target of selective serotonin reuptake inhibitors (SSRIs). Indeed, these drugs had no effect on *Trek1*^−/−^ mice (Heurteaux et al., 2006). Accordingly, Trek1 represents a potential target for novel therapeutic strategies to combat depression (Perlis et al., 2008; Liou et al., 2009; Mazella et al., 2010; Moha ou Maati et al., 2011; Moha Ou Maati et al., 2012).

Compared with *Trek1*, relatively little is known about the neurobiological relevance of *Trek2*. An early study described a limited CNS distribution of *Trek2* (Talley et al., 2001). Other evidence, however, including results from human tissue PCR, *in situ* hybridization from zebrafish, and rat *in situ* hybridization data suggest that *Trek2* is expressed broadly throughout the CNS (Medhurst et al., 2001; Gu et al., 2002; Gierten et al., 2012). Moreover, we reported recently using a cDNA panel that *Trek2* mRNA is expressed in most regions of the mouse brain (Mirkovic and Wickman, 2011). These observations suggest that *Trek2* may make a broad and significant contribution to neurophysiology and behavior.

While RNAi-dependent knockdown of *Trek2* in the entorhinal cortex was shown to disrupt spatial memory (Deng et al., 2009), data concerning the neurobehavioral relevance of *Trek2* are scant. The recent development of mice lacking *Trek2* (*Trek2*^−/−^) and all three members of the Trek channel family (*Trek1/2/Traak*^−/−^), however, permit rigorous testing of the role of Trek channels, and Trek2-containing channels in particular, in behavior (Heurteaux et al., 2004; Guyon et al., 2009). In this study, we probe the consequences of constitutive *Trek* gene ablation in mice, in paradigms that assess motor activity, coordination, anxiety-related behavior, learning and memory, and drug-induced reward-related behavior.

## Materials and methods

### Experimental subjects

All animal use was approved by the University of Minnesota Institutional Animal Care and Use Committee and carried out in accordance with National Institutes of Health guidelines. All mice used in this study were bred on-site, housed in same-sex groups of 2–5 after weaning, and provided with food and water *ad libitum*. Mice were kept on a 12 h light/dark cycle, with lights on between 0700 and 1900. All tests were performed between 0900 and 1600. Mice lacking *Trek2* (*Trek2*^−/−^) and mice lacking *Trek1, Trek2, and Traak* (*Trek1/2/Traak*^−/−^) were generated as described (Heurteaux et al., 2004; Guyon et al., 2009). Null mutations were backcrossed against the C57BL/6J inbred strain for 10+ generations prior to establishing the breeding cages used to generate subjects for this study. Both male and female mice (5–10 weeks) were evaluated in all behavioral tests. No more than three distinct behavioral tests were performed on any single animal, and in no instance were animals evaluated in any test after completing the morphine-induced motor activity, conditioned place preference (CPP) studies, or the contextual fear conditioning test.

### Locomotor activity

One day prior to locomotor activity assessments, mice were habituated to handling (5 min) and testing room (60 min). On the first day of testing, mice were placed in open-field activity chambers (ENV-515; Med Associates, Inc.; St. Albans, VT), housed within sound-attenuating cubicles for 60 min. The open-field was illuminated with 28 V DC/100 mA house lights (ENV-215 M; Med Associates, Inc.) during testing. Total distance traveled, thigmotaxis, and time/entries into the central area of the open-field were recorded using Open Field Activity Software package v. 4.0 (Med Associates, Inc.). For the morphine-induced motor activity study, total distance traveled was measured for 60 min, beginning 10 min after an intraperitoneal (i.p.) injection of morphine (Sigma; St Louis, MO). Each mouse received all morphine doses, with three rest days between injections.

### Rotarod

Motor coordination was assessed using an accelerating rotarod (IITC Life Sciences; Woodland Hills, CA), as described (Anderson et al., 2010). Briefly, mice were acclimated to the testing room 1 h prior to evaluation. Each subject was given two trials to acclimate to the task, followed by 6-test trial. Animals were allowed a minimum of 15 min to rest between trials, followed by a 2 h break after trial 4. After placement of the subject on the rod, the rod was accelerated from 4 to 27 rpm over a 5 min period. Latency to fall was recorded when a subject fell from the rod, or made 2 full revolutions while clinging to the drum.

### Elevated plus maze

Anxiety-related behavior was measured using the elevated plus maze, as described (Pravetoni and Wickman, 2008). In brief, mice were acclimated to the testing room 1 h prior to evaluation. The maze consisted of two open and two closed arms, as well as an exposed center panel, elevated 52 cm off the floor of the testing room (Columbus Instruments, Inc.; Columbus, OH). Testing was conducted under standard room lighting conditions. Each trial began with the placement of the mouse in the maze center, facing a closed arm; subsequent activity was recorded by video camera for 5 min. The time spent by each mouse in the open and closed arms was scored manually by two investigators blind to subject genotype. Time spent in the EPM center was not included as time in either open or closed arms.

### Light/dark box

The light/dark test was performed in a modified two-compartment mouse CPP chamber (Env-3013-2; Med Associates, Inc.) housed in a sound-attenuating cubicle. Flooring was normalized in both compartments using plexiglass inserts. The overhead light was turned off in the black chamber (dark), while the white (light) chamber was illuminated by a 28 V DC/100 mA light bulb (ENV-221M; Med Associates, Inc.) connected to a 3-channel light control unit (ENV-226B; Med Associates, Inc.) set at intensity level 10. Mice were acclimated to the testing room for 30 min before testing. The mouse was placed in the center of the black chamber and allowed full access to both compartments after a 5 s delay. Time spent and distance traveled in both compartments was recorded during a 10 min trial using Med-PC software (Med Associates, Inc.).

### Contextual fear conditioning

Contextual fear conditioning was conducted in 30.5 × 24.1 × 21.0 cm conditioning chambers (VFC_008; Med Associates, Inc.), housed in a sound-attenuating cubicle, utilizing steel bar flooring connected to a shock generator. The chamber was illuminated using a combination infrared and visual overhead light box (NIR-100; Med Associates, Inc.). Mice were allowed to acclimate to the testing room for 30 min prior to testing. Training consisted of 120 s baseline exposure followed by three conditioning trials. Each conditioning trial consisted of a 20 s light cue (ENV-229M; Med Associates, Inc.), a 20 s latency period, and a 2 s shock (0.7 mA), with an inter-trial interval of 60 s. A 5 min test session was conducted 24 h later, where time spent freezing and number of freezing episodes were recorded using “Video freeze” software (Med Associates, Inc.).

### Novel object recognition

The novel object recognition study was performed using open-field environments (ENV-022MD; Med Associates, Inc.), housed within sound-attenuating cubicles, and illuminated with 28 V DC/100 mA house lights (ENV-215M; Med Associates, Inc.). On Day 1, mice were handled and habituated to the open field environment for 60 min. On Day 2, mice were evaluated over four sessions, separated by 10 min breaks, during which subjects were returned to their home cages. In Session 1, animals were allowed to explore the open-field environment for 30 min. In Sessions 2 and 3, mice were re-introduced to the open-field environment for 10 min, and in both sessions the environments contained two identical objects (familiar). In Session 4, mice were re-introduced to the open-field environment for 5 min, now containing one familiar and one novel object. Session 4 was videotaped and the time subjects spent interacting with each object was recorded manually. A subject was considered to be interacting with an object if its nose was directed toward (and within 2 cm of) the object. Objects used in this study were a single red Duplo (The LEGO Group; Enfield, CT) and a blue cap from a 50 mL conical tube. Objects were counter-balanced to account for any object preference effects.

### Conditioned place preference

CPP was performed in two-compartment mouse CPP chambers (Env-3013-2; Med Associates, Inc.), housed within sound-attenuating cubicles. One compartment contained white walls and a mesh floor, and the other contained black walls and a rod floor. The overhead light was identical in both chambers: a 28 V DC/100 mA light (ENV-221M; Med Associates, Inc.) connected to a 3-channel light control unit (ENV-226B; Med Associates, Inc.) set at intensity level 8. All subjects were acclimated to handling (5 min) and the testing room (1 h) for 2–3 d prior to beginning the 5-day test. On Day 1, mice were placed in the chamber for 15 min with the door separating compartments open; time spent in each compartment was recorded. Subjects spending more than 65% of their time in either compartment on Day 1 were excluded from the study. On Days 2–4, mice were subjected to two 20-min conditioning sessions, one in the morning (0900–1100) and one in the afternoon (1400–1600). In the morning session, mice were given a subcutaneous (s.c.) saline injection and confined to the compartment that was preferred on Day 1. In the afternoon sessions, mice were given s.c. morphine and confined to the opposite compartment. On Day 5, mice were placed in the chamber for 15 min with the door separating the compartments open; time spent in each compartment was then recorded during a 15 min test session using Med-PC software. The change in time spent on the drug-paired side as measured on Day 1 and Day 5 (Day 5—Day 1) was taken as the measure of morphine-induced CPP.

### Statistical analysis

Data are expressed throughout as mean ± SEM and were analyzed using Sigma Plot 11.0 (Systat Software Inc.; Chicago, IL) or Prism v. 11 (GraphPad Software Inc.; La Jolla, CA). The potential impact of gender on performance in each task was assessed with two-way ANOVA for all tasks except rotarod, morphine-induced locomotor activation, and CPP, where a 3-way ANOVA was used. If the results of statistical analysis revealed no significant impact of gender on task performance, data from male and female subjects were pooled to increase the power of the analysis. Open field activity, elevated plus maze, light/dark box, contextual fear conditioning, and novel object recognition data were analyzed using one-way ANOVA; the Newman Keuls *post hoc* test was used when a significant interaction was found. For rotarod and morphine-induced motor activity data, a two-way ANOVA with repeated measures was used. Data from the morphine-induced CPP study was analyzed using a standard two-way ANOVA. Differences were considered significant if *P* < 0.05.

## Results

The main goal of this study was to evaluate wild-type C57BL/6J and congenic *Trek2*^−/−^ mice in tests of motor activity, coordination, anxiety, learning and memory, and reward-related behavior. Congenic mice lacking all three members of the Trek subfamily (*Trek1/2/Traak*^−/−^ mice) were evaluated in parallel, as up-regulation of *Trek1* and/or *Traak* might compensate for the loss of *Trek2* and suppress neurobehavioral phenotypes. Prior to testing, we profiled *Trek2*^−/−^ and *Trek1/2/Traak*^−/−^ mice for gross deficiencies in sensory perception that might influence performance in the chosen behavioral tests. *Trek2*^−/−^ and *Trek1/2/Traak*^−/−^ mice were indistinguishable from wild-type counterparts by all visual criteria, and they responded normally, without excessive vocalization, to gentle prodding and handling. No genotype-dependent differences in body weight were observed (not shown). Following acclimation to a dark room, *Trek2*^−/−^ and *Trek1/2/Traak*^−/−^ mice exhibited a normal pupil response (constriction) to a bright light. *Trek2*^−/−^ and *Trek1/2/Traak*^−/−^ mice also exhibited normal forepaw extension upon lowering to their home cage or benchtop, indicative of intact vision and normal depth perception. *Trek2*^−/−^ and *Trek1/2/Traak*^−/−^ mice exhibited normal responses to sudden sounds (clapping), arguing against gross deficits in hearing. Thus, no obvious developmental abnormalities, or deficits in touch, vision, or hearing, were evident in *Trek2*^−/−^ and *Trek1/2/Traak*^−/−^ mice.

### Motor activity and coordination

**Figure 1:**
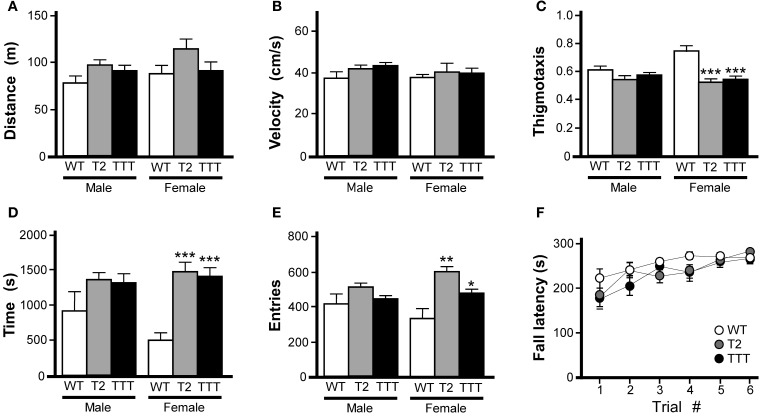
**Motor activity and coordination in mice lacking *Trek* channels.** Open-field activity was measured in wild-type (WT, white), *Trek2^−/−^* (T2, gray), and *Trek1/2*/Traak^−/−^ (TTT, black) mice during a 60 min test session (*n* = 8–12 per group). **(A)** Total distance traveled during the 60 min session. No genotype-dependent differences were observed between male [*F*_(2, 29)_ = 2.3, *P* = 0.12] or female [*F*_(2, 30)_ = 1.9, *P* = 0.17] *Trek2*^−/−^, *Trek1/2/Traak*^−/−^, and wild-type mice. **(B)** Velocity during open-field activity; no genotype-dependent differences were observed between male [*F*_(2, 29)_ = 2.1, *P* = 0.14] or female [*F*_(2, 30)_ = 0.3, *P* = 0.78] *Trek2*^−/−^, *Trek1/2/Traak*^−/−^, and wild-type mice. **(C)** Thigmotaxis scores (distance traveled in periphery/total distance traveled); no genotype-dependent differences were observed in male animals [*F*_(2, 29)_ = 2.2, *P* = 0.13], whereas both *Trek2*^−/−^ and *Trek1/2*/Traak^−/−^ females traveled less distance in the field periphery than wild-type controls [*F*_(2, 30)_ = 11.0, *P* < 0.001]. **(D)** Time spent in the center of the open-field; a mild genotype-dependent difference was observed for male animals [*F*_(2, 29)_ = 3.4, *P* < 0.05], though *post hoc* pairwise comparisons did not reveal a difference between *Trek2*^−/−^, *Trek1/2*/Traak^−/−^, and wild-type controls. *Trek2*^−/−^ and *Trek1/2*/Traak^−/−^ females spent significantly more time in the center than wild-type controls [*F*_(2, 30)_ = 18.7, *P* < 0.0001]. **(E)** Number of entries into center of the open-field; no genotype-dependent differences were observed in male animals [*F*_(2, 29)_ = 2.1, *P* = 0.14], whereas both *Trek2*^−/−^ and *Trek1/2*/Traak^−/−^ females made more entries into the center than wild-type controls [*F*_(2, 30)_ = 7.7, *P* < 0.01]. **(F)** Rotarod performance of wild-type, *Trek2*^−/−^, and *Trek1/2*/Traak^−/−^ mice, measured over six separate trials (*n* = 11–18 per group). A main effect of trial number was observed [*F*_(5, 228)_ = 8.80, *P* < 0.001]; within-genotype, pair-wise comparisons are not shown on the plot. A main effect of genotype was not detected [*F*_(2, 228)_ = 3.0, *P* = 0.06], nor was there a significant interaction between trial and genotype [*F*_(10, 228)_ = 0.8, *P* = 0.62]. Symbols: ^*^,^**^,^***^ *P* < 0.05, 0.01, and 0.001, respectively, vs. wild-type.

Wild-type, *Trek2*^−/−^ and *Trek1/2/Traak*^−/−^ mice were monitored for 60 min in open-field activity chambers for distance traveled and position within the field. Because we observed an influence of gender on time spent in and entries into the center of the open-field, all open-field activity data for male and female subjects were analyzed separately. No genotype-dependent differences were observed in male or female mice with respect to total distance traveled (Figure 1A) or velocity (Figure 1B). In male mice, no genotype-dependent differences were detected with respect to thigmotaxis (calculated as the ratio of distance traveled in the field periphery to total distance traveled), time spent in the center of the open-field, or number of entries into the center of the open-field (Figures 1C-E). In female mice, however, both *Trek2*^−/−^ and *Trek1/2/Traak*^−/−^ mice showed less thigmotaxis (Figure 1C). Female *Trek2*^−/−^ and *Trek1/2/Traak*^−/−^ mice also spent more time than wild-type controls in the field center (Figure 1D), and exhibited significantly more entries into the center as compared to wild-type female controls (Figure 1E), behaviors consistent with lower anxiety-related behavior (Simon et al., 1994).

Motor coordination was evaluated in wild-type, *Trek2*^−/−^, and *Trek1/2/Traak*^−/−^ mice using an accelerating rotarod test as described (Anderson et al., 2010). No effect of gender was observed in this task [*F*_(1, 263)_ = 2.9, *P* = 0.09]; as such male and female data were pooled. No genotype-dependent differences were observed with respect to ability of the mice to learn the task, learning rate, or peak performance (Figure 1F).

### Anxiety-related behavior

**Figure 2:**
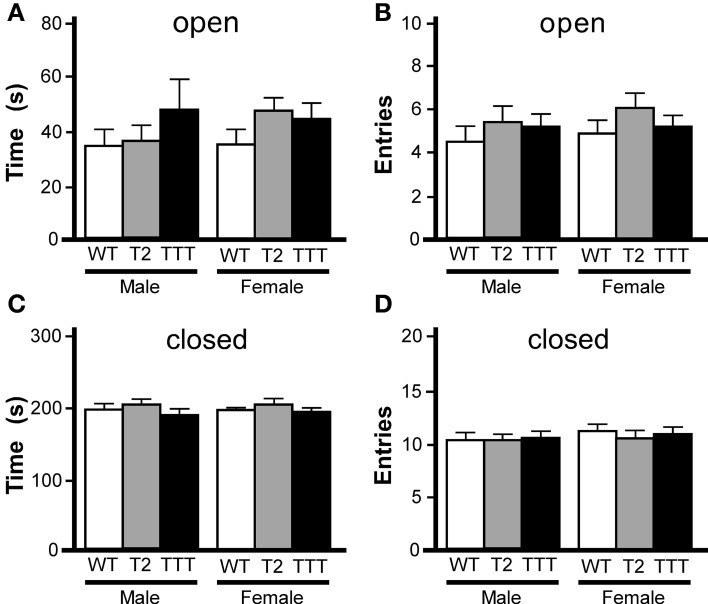
**Elevated plus maze performance in mice lacking *Trek* channels.** Wild-type (WT, white), *Trek2*^−/−^ (T2, gray), and *Trek1/2*/Traak^−/−^ (TTT, black) mice were evaluated in a 5 min (300 s) EPM test (*n* = 8–19 per group). No genotype-dependent differences were observed with respect to male or female mice in time spent in **(A)** [male: *F*_(2, 40)_ = 0.8, *P* = 0.46; female: *F*_(2, 49)_ = 1.4, *P* = 0.25] or number of entries into **(B)** [male: *F*_(2, 40)_ = 1.9, *P* = 0.16; female: *F*_(2, 49)_ = 0.9, *P* = 0.41] the open arms. Likewise, no significant differences were observed with respect to genotype in time spent in **(C)** [male: *F*_(2, 40)_ = 0.5, *P* = 0.59; female: *F*_(2, 49)_ = 0.1, *P* = 0.93] or number of entries into **(D)** [male: *F*_(2, 40)_ = 0.03, *P* = 0.97; female: *F*_(2, 48)_ = 0.3, *P* = 0.77] the closed arms.

While rotarod and open-field activity data indicated that *Trek2*^−/−^ and *Trek1/2/Traak*^−/−^ mice do not exhibit gross deficiencies in motor activity or coordination, the reduced thigmotaxis observed in female *Trek2*^−/−^ and *Trek1/2/Traak*^−/−^ mice argued that Trek channels may influence anxiety-related behavior. To gain additional insight into anxiety-related behavior in *Trek2*^−/−^ and *Trek1/2/Traak*^−/−^ mice, we next examined performance in an elevated plus maze, an established measure of anxiety-related behavior (Lister, 1987). While *Trek2*^−/−^ and *Trek1/2/Traak*^−/−^ mice tended to spend more time in the open arms (Figure 2A), and made more entries into the open arms of the maze (Figure 2B), differences were not significant, for either gender. Similarly, no genotype-dependent differences were observed in either male or female mice in terms of the time spent in closed arms (Figure 2C) or number of entries into the closed arms (Figure 2D).

**Figure 3:**
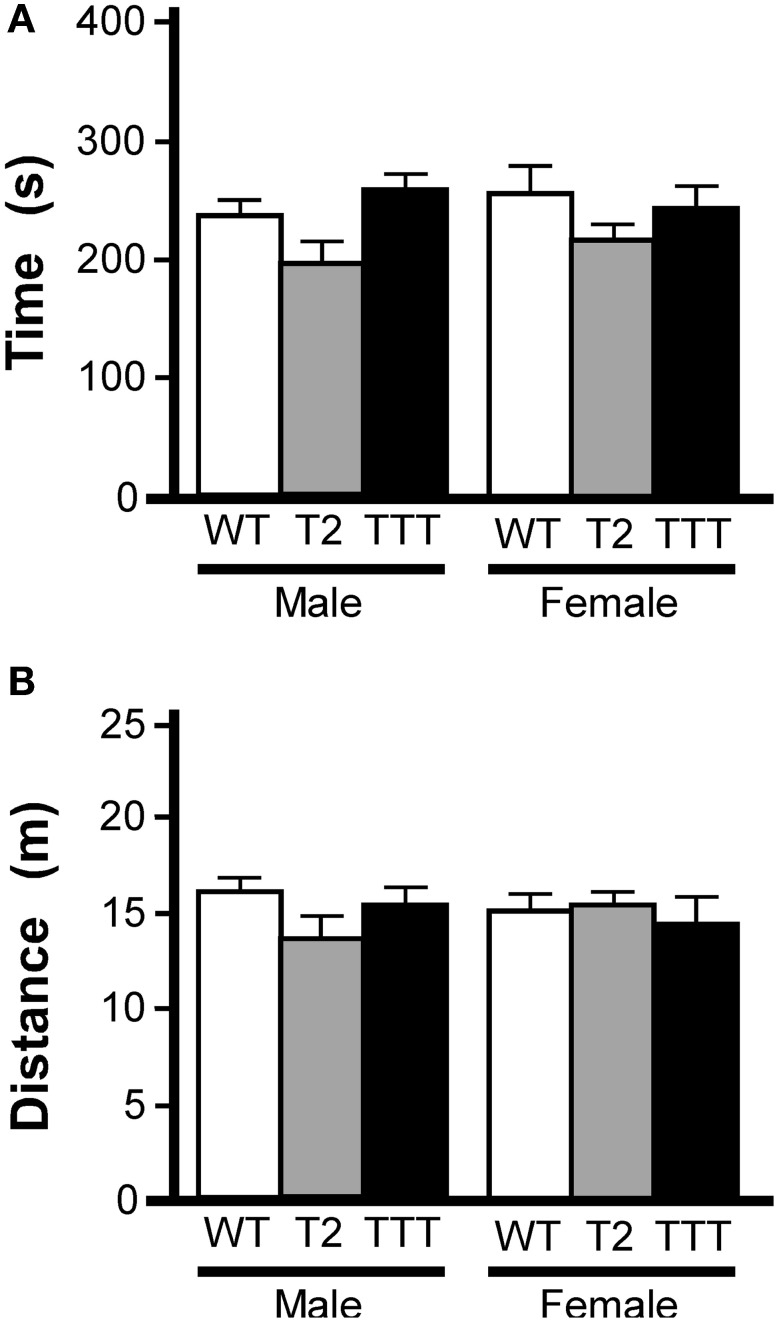
**Light/dark box behavior in mice lacking *Trek* channels.** Wild-type (WT, white), *Trek2*^−/−^ (T2, gray), and *Trek1/2*/Traak^−/−^ (TTT, black) mice were evaluated in a 10 min (600 s) light/dark box test (*n* = 5–12 per group). **(A)** Time spent in the light compartment; no genotype-dependent differences were observed for male [*F*_(2, 22)_ = 2.7, *P* = 0.09] or female [*F*_(2, 25)_ = 1.0, *P* = 0.38] mice. **(B)** Distance traveled in the light compartment; no genotype-dependent differences were observed in male [*F*_(2, 22)_ = 1.7, *P* = 0.21] or female [*F*_(2, 25)_ = 0.2, *P* = 0.82] mice.

We also tested animals in the light/dark box, an alternative measure of anxiety-related behavior (Bourin and Hascoet, 2003). In this task, animals are placed in a two-compartment chamber, one dark and the other brightly illuminated. Increased time spent in the light chamber is consistent with reduced anxiety-related behavior (Costall et al., 1989). As an influence of gender was previously observed in the open-field test, we again analyzed data from male and female subjects separately. Total time spent in the light compartment did not differ between genotypes in male or female mice (Figure 3A). Moreover, no gender or genotype differences were observed with respect to total distance traveled during the 10-min trial (not shown) or distance traveled in the light compartment (Figure 3B).

### Learning and memory

**Figure 4:**
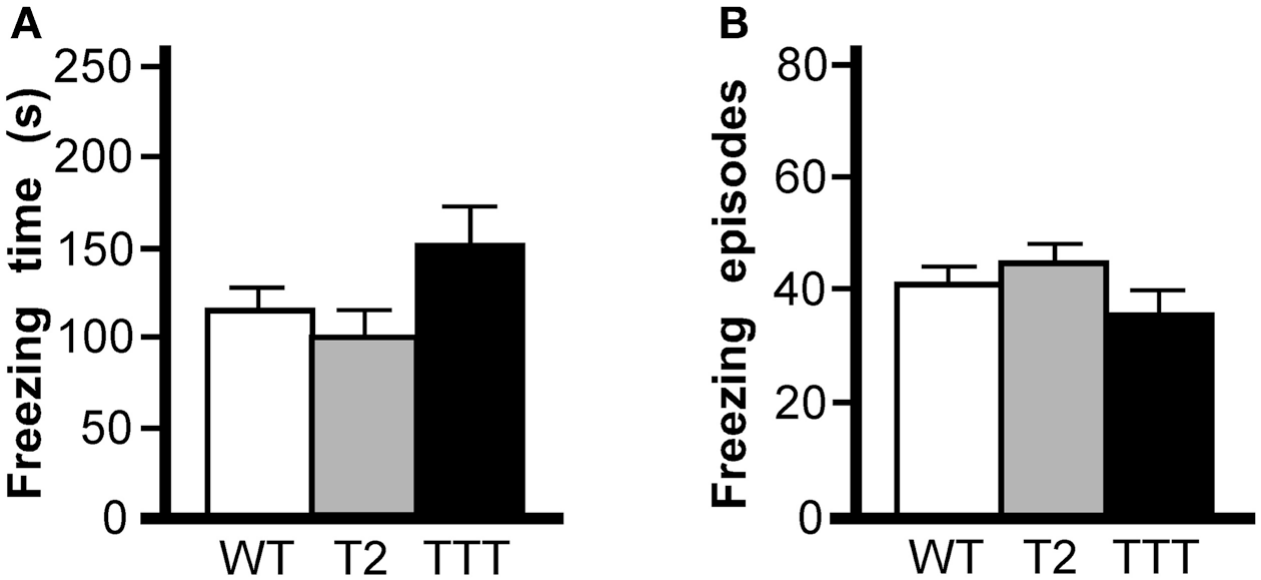
**Contextual fear conditioning in mice lacking *Trek* channels.** Wild-type (WT, white), *Trek2*^−/−^ (T2, gray), and *Trek1/2*/Traak^−/−^ (TTT, black) mice were evaluated in 5 min (300 s) contextual fear conditioning test (*n* = 9–14 per group). **(A)** Time spent freezing; no genotype-dependent differences were observed with respect to time spent freezing [*F*_(2, 34)_ = 2.4, *P* = 0.11]. **(B)** Freezing episodes; no genotype-dependent differences were observed with respect to number of freezing episodes [*F*_(2, 34)_ = 1.4, *P* = 0.25].

To assess the learning and memory ability of *Trek2*^−/−^ and *Trek1/2/Traak*^−/−^ mice, we first tested animals in a contextual fear conditioning task. This 2 d test of Pavlovian learning involves the association of a painful stimulus (foot shock) with an environment (Rudy et al., 2004). The first session included three conditioning trials separate by 60 s, each consisting of a 2 s (0.7 mA) shock delivered after presentation of a 20 s light cue. The test day consisted of a 5 min re-exposure to the training environment and evaluation of freezing behavior. No effect of gender was observed in total time spent freezing [*F*_(1, 29)_ = 2.8, *P* = 0.11] or in the number of freezing episodes [*F*_(1, 29)_ = 0.1, *P* = 0.72]; as such, data from male and female subjects were pooled. No genotype-dependent differences were observed in total time spent freezing (Figure 4A) or in the number of freezing episodes (Figure 4B).

**Figure 5:**
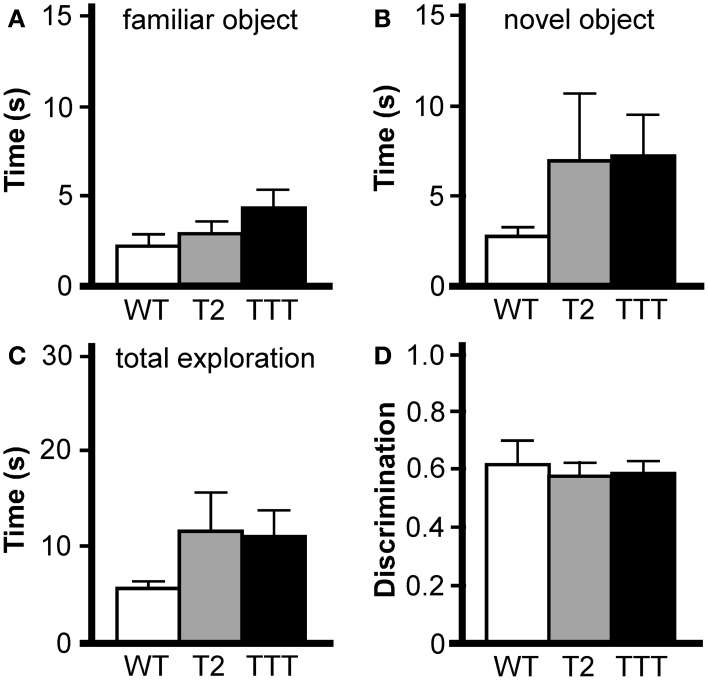
**Novel object recognition in mice lacking *Trek* channels.** Wild-type (WT, white), *Trek2*^−/−^ (T2, gray), and *Trek1/2*/Traak^−/−^ (TTT, black) mice were evaluated in a novel object recognition task (*n* = 7–9 per group). No genotype-dependent differences were observed in terms of the amount of time spent during a 5 min (300 s) test session interacting with the familiar object **(A)** [*F*_(2, 22)_ = 1.7, *P* = 0.21], time spent interacting with the novel object **(B)** [*F*_(2, 22)_ = 1.0, *P* = 0.39], total time interacting with the familiar and novel object **(C)** [*F*_(2, 22)_ = 1.2, *P* = 0.31], or in object discrimination ratio **(D)** [*F*_(2, 22)_ = 0.1, *P* = 0.87], defined as the ratio of time spent exploring the novel object to the total time spent exploring both objects [time_novel_/(time_familiar_ + time_novel_)].

We next tested the effect of *Trek* ablation in the novel object recognition task, which has been used to assess working memory, anxiety, and preference for novelty in rodents (Dere et al., 2007). The task requires an animal to recognize and recall prior experience with a familiar object, and discriminate that object from a novel object. In our paradigm, mice were exposed to the open-field chamber for 60 min on the day prior to testing. On test day, mice were re-introduced to the open-field chamber for 30 min, then again in two consecutive 10 min sessions incorporating an object (familiar), and finally in a 5-min test session where both the familiar object and a novel object were present in the field. The time spent exploring both objects were recorded during the test session. No effect of gender was observed for any parameter analyzed (time exploring novel object, *F*_(1, 19)_ = 0.4, *P* = 0.55; time exploring familiar object, *F*_(1, 19)_ = 0.8, *P* = 0.39; total time exploring objects, *F*_(1, 19)_ = 0.1, *P* = 0.78; discrimination ratio, *F*_(1, 19)_ = 2.3, *P* = 0.14), and as such, data from male and female subjects were pooled. The total time spent exploring both the familiar (Figure 5A) and novel (Figure 5B) objects during the test session were not different across genotypes, nor was the total time spent exploring both objects (Figure 5C). And, while all genotypes showed a preference for the novel object over the familiar, no differences were observed with respect to genotype in this regard (Figure 5D).

### Reward-related behavior

**Figure 6:**
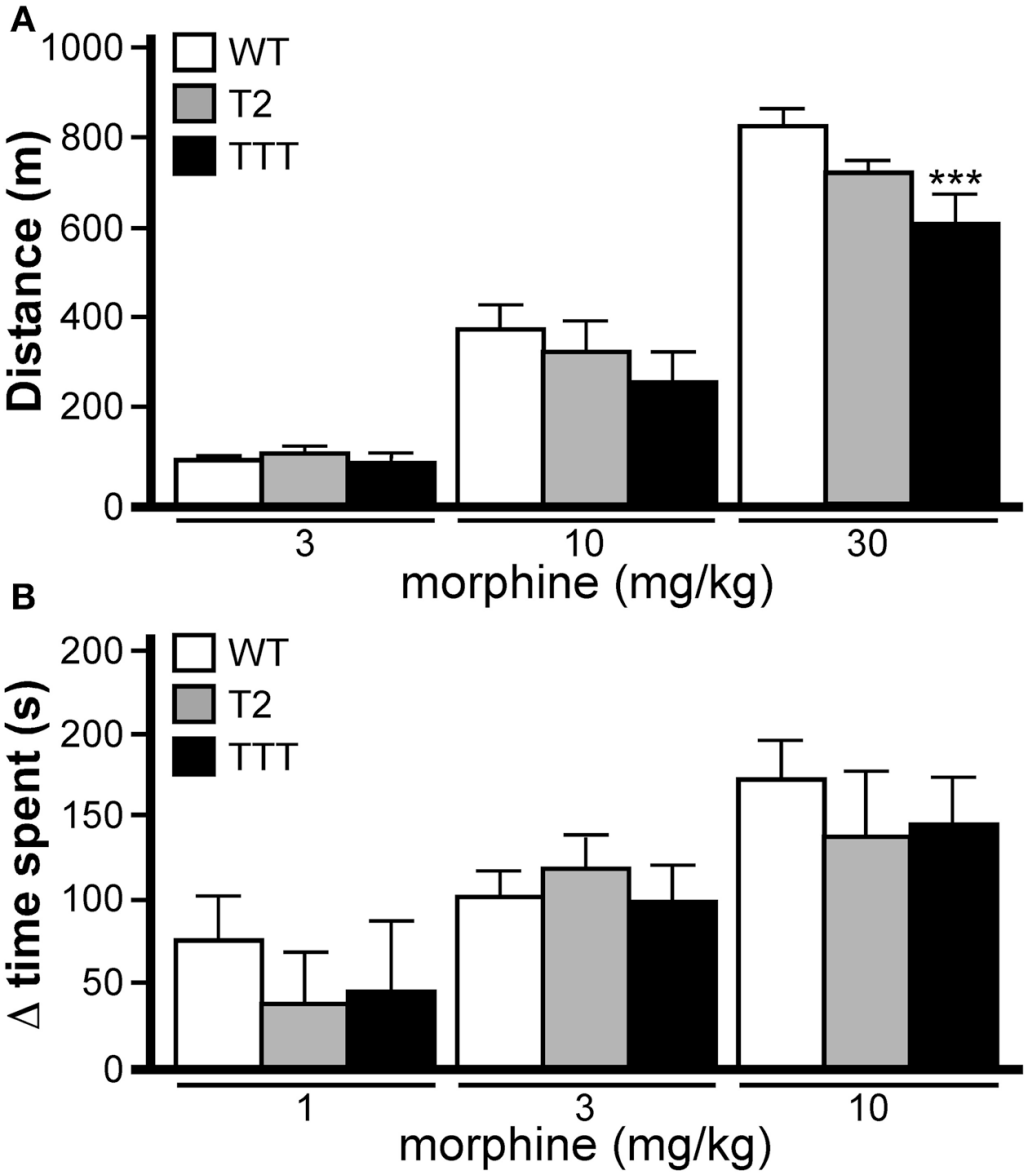
**Opioid-induced motor activity and reward in mice lacking *Trek* channels. (A)** Morphine-induced locomotor activity was measured during a 60 min session in wild-type (WT, white), *Trek2*^−/−^ (T2, gray), and *Trek1/2*/Traak^−/−^ (TTT, black) mice (*n* = 12–16 per group). Significant main effects of morphine dose [*F*_(3, 144)_ = 250.6, *P* < 0.0001] and genotype [*F*_(2, 144)_ = 4.6, *P* < 0.05] were observed, as well as a dose x genotype interaction [*F*_(6, 114)_ = 2.5, *P* < 0.05]. While no significant differences were observed between genotypes at either the 3 mg/kg or 10 mg/kg doses, *Trek1/2*/Traak^−/−^ showed a slightly blunted response to 30 mg/kg morphine as compared to wild-type controls. (**B)** Morphine-induced CPP was measured in wild-type (*n* = 10–15/dose), *Trek2*^−/−^ (*n* = 7–9/dose), and *Trek1/2*/Traak^−/−^ (*n* = 6–7/dose) mice using a standard CPP test. While a significant main effect of morphine dose was observed [*F*_(2, 73)_ = 9.0, *P* < 0.001], there was no main effect of genotype [*F*_(2, 73)_ = 0.5, *P* = 0.59] or dose x genotype interaction [*F*_(4, 73)_ = 0.32, *P* = 0.86]. No genotype-dependent differences were observed in morphine-induced CPP, as measured by calculating the change in time spent (Δ time spent) in the drug-paired side from Day 1–5. In panels **A** and **B**, only within-dose pair-wise comparisons are highlighted. Symbols: ^***^ *P* < 0.01, vs. wild-type.

Normal opioid-induced motor stimulation and CPP require activation of mu opioid receptors in midbrain structures, including the ventral tegmental area (Bozarth and Wise, 1986; Bozarth, 1987; Kalivas and Duffy, 1987; Latimer et al., 1987). Mu opioid receptors are metabotropic (G protein-coupled) receptors linked to the G_i/o_ subclass of G proteins. Previous studies have indicated that both Trek1 and Trek2 can be activated by GPCRs linked to G_i/o_ G proteins (Fink et al., 1996; Patel et al., 1998; Lesage et al., 2000; Murbartian et al., 2005; Deng et al., 2009; Xiao et al., 2009), and both channels are expressed in the mouse midbrain (Lein et al., 2007). Accordingly, we evaluated the impact of *Trek* ablation on the motor-stimulatory and rewarding effect of morphine, the prototypical mu opioid receptor agonist. Morphine doses higher than 30 mg/kg yielded elevated stereotypic movements and reduced overall activity levels in wild-type mice (not shown), and thus, 30 mg/kg morphine was selected as the maximal dose in this study. No effect of gender was observed in this task [*F*_(1, 114)_ < 0.001, *P* = 0.98]; as such, data from male and female subjects were pooled. Systemic morphine administration stimulated motor activity in a dose-dependent manner in all genotypes (Figure 6A). While no difference between genotypes was observed at the lowest morphine doses evaluated (3 and 10 mg/kg), *Trek1/2/Traak*^−/−^ mice exhibited less morphine-induced motor activity at the 30 mg/kg dose.

Morphine-induced CPP was analyzed in wild-type, *Trek2*^−/−^ and *Trek1/2/Traak*^−/−^ mice using a two-compartment chamber. On Day 1, mice were allowed to explore both sides of the chamber during a 15 min session, and time spent on each side of the chamber was recorded. Over the next 3 days, animals were subjected to conditioning sessions where saline (AM session) or morphine (1, 3, or 10 mg/kg; PM session) was administered systemically prior to confinement in a defined (counterbalanced) side of the chamber. In a pilot study involving wild-type mice, no difference in the magnitude of the morphine-induced CPP was observed for 10 and 30 mg/kg morphine doses [*t*_22_ = 0.2, *P* = 0.9]. Accordingly, wild-type and *Trek*^−/−^ mice were challenged with 10 mg/kg morphine as the highest dose in this test. No effect of gender was observed [*F*_(1, 64)_ = 0.1, *P* = 0.81]; as such, male and female data was pooled. A dose-dependent increase in time spent in the drug-paired side was observed for all genotypes, and the magnitude of the morphine-induced CPP measured in *Trek2*^−/−^ and *Trek1/2/Traak^−/−^* mice was no different from wild-type controls at any dose tested (Figure 6B).

## Discussion

Recent work by our laboratory, as well as *in situ* hybridization data collected by the Allen Institute for Brain Research (Lein et al., 2007), suggests a widespread distribution of *Trek2* mRNA in the mouse CNS. Accordingly, and given the well-documented and broad expression of *Trek1* and *Traak* in the rodent CNS (Medhurst et al., 2001; Talley et al., 2001; Gu et al., 2002), one might predict that Trek channel activity influences neurophysiology and behavior in a broad manner. The primary goal of this study was to begin assessing the impact of *Trek* ablation on mouse behavior, using a representative battery of behavioral paradigms.

*Trek* gene ablation exerted little influence on motor activity or coordination. A significant difference, and clear interaction between genotype and gender, was observed in anxiety-related behavior in the open-field. Female *Trek2^−/−^* and *Trek1/2/Traak^−/−^* mice exhibited significantly reduced thigmotaxis and increased time spent in the center of the open-field. While many brain regions have been linked to anxiety, the hippocampus and amygdala are key anatomic loci for anxiety-related behavior (Menard and Treit, 1999; Canteras et al., 2010). In this regard, the relatively high level of *Trek2* mRNA observed in both structures in the mouse and human may be relevant (Medhurst et al., 2001; Lein et al., 2007; Mirkovic and Wickman, 2011). However, *Trek2^−/−^* and *Trek1/2/Traak^−/−^* mice performed similar to wild-type controls in the elevated plus maze and light/dark box tests, other established tests of anxiety-related behavior. Such discrepancies within anxiety-related behavioral paradigms have been reported for other mutant mouse strains (Salas et al., 2003; Bhatnagar et al., 2004; Lau et al., 2008), and argue that any influence of Trek channels on anxiety-related behavior is modest and/or not uniform.

Recent work has shown that Trek channels mediate, in part, the inhibitory effect of GABA_B_ receptor stimulation on neurons in the hippocampus and entorhinal cortex (Deng et al., 2009; Sandoz et al., 2012), and have suggested a role for *Trek* channels in learning and memory (Deng et al., 2009). We, however, observed no influence of *Trek* ablation on learning and memory, as assessed using novel object recognition and contextual fear conditioning tasks. One concern in studies involving these and other standard paradigms is whether underlying abnormalities in sensory perception influence behavioral outcomes. And while our experience did not reveal gross deficiencies in vision, hearing, or touch in *Trek*^−/−^ mice, we cannot exclude the possibility that subtle differences exist that impacted their performance in some of the chosen tasks. For example, previous studies have revealed decreased pain thresholds with *Trek1*^−/−^ and *Traak*^−/−^ mice (Alloui et al., 2006; Noel et al., 2009). As such, the behavior of *Trek*^−/−^ mice in paradigms that involve an aversive stimulus, such as a foot shock (e.g., contextual fear conditioning), could reflect altered sensitivity to the aversive stimulus, altered associative learning processes and/or memory recall, or a mixture of influences. Nevertheless, the simplest interpretation of our fear conditioning and novel object recognition data is that Trek channels exert little if any significant influence over associative learning processes.

Trek channels are activated by receptors linked to G_i/o_ G proteins in a process thought to involve inhibition of cAMP production and a decrease in PKA-dependent phosphorylation (Patel et al., 1998; Lesage et al., 2000; Murbartian et al., 2005; Deng et al., 2009; Xiao et al., 2009). Opioids such as morphine bind to G_i/o_ coupled receptors expressed in the midbrain, leading to increased motor activity and CPP (Bozarth and Wise, 1986; Bozarth, 1987; Kalivas and Duffy, 1987; Latimer et al., 1987). As *Trek* channels are expressed in the mouse midbrain (Lein et al., 2007), we probed for a contribution of Trek channels to opioid-induced reward-related behavior. Interestingly, *Trek1/2/Traak^−/−^* mice did display a blunted motor-stimulatory response to the highest dose of morphine tested. As *Trek2^−/−^* mice behaved normally in this task, the phenotype is most likely attributable to the loss of *Trek1*, since Traak channels are not modulated by G protein signaling (Fink et al., 1998; Maingret et al., 2000; Kim et al., 2001). In contrast, we found no impact of Trek channel ablation on morphine-induced CPP. Thus, consistent with our assessment of anxiety-related behavior in *Trek*^−/−^ mice, any contribution of Trek channels to opioid-induced reward-related behavior appear to be modest and paradigm-dependent.

While the broad CNS distribution of Trek channels suggests that this channel family makes significant contributions to many behaviors, we found few neurobehavioral phenotypes in *Trek*^−/−^ mice in this study. An important consideration for any study involving constitutive gene ablation is that subtle molecular and/or developmental compensation might mask the influence of a gene or genes on complex behaviors. Accordingly, data from studies involving constitutive knockout mice should be interpreted with caution until corroborated by data obtained via alternative approaches. This is particularly true for studies such as this, where the behavior of multiple congenic mutant lines is compared to a single wild-type control group rather than to same-sex wild-type littermates. Nevertheless, the simplest interpretation of the available data is that the neurobehavioral influence of Trek channels is relatively modest or non-existent, at least in the context of the behaviors evaluated in this study.

One intriguing scenario that might reconcile the broad CNS distribution of *Trek* channels with their modest impact on behavior is that Trek channel activity is low under normal conditions, becoming evident and impactful only under certain circumstances, such as ischemia (Heurteaux et al., 2004; Buckler and Honore, 2005; Caley et al., 2005; Wang et al., 2012). Indeed, Trek channels are activated by polyunsaturated fatty acids (Patel et al., 1998; Lesage et al., 2000; Maingret et al., 2000), intracellular acidification (Maingret et al., 1999; Lesage et al., 2000; Kim et al., 2001), and membrane stretch (Patel et al., 1998; Bang et al., 2000; Lesage et al., 2000), and all three of these influences are triggered by cerebral ischemia. Moreover, *Trek1*^−/−^ mice show enhanced sensitivity to ischemia (Heurteaux et al., 2004), and *Trek1* is up-regulated after focal ischemia (Wang et al., 2012). Recently, *Trek2* expression was shown to increase after exposure to ischemic conditions (Kucheryavykh et al., 2009). Thus, future studies exploring the neurophysiological relevance of Trek2-containing channels under ischemic conditions will be important.

### Conflict of interest statement

The authors declare that the research was conducted in the absence of any commercial or financial relationships that could be construed as a potential conflict of interest.
